# Exploring the role of Peanut (*Arachis hypogaea* L.) root architecture in enhancing adaptation to climate change for sustainable agriculture and resilient crop production: A review

**DOI:** 10.1016/j.jgeb.2025.100535

**Published:** 2025-07-11

**Authors:** Yohannes Gelaye, Jihua Li, Huaiyong Luo

**Affiliations:** a Key Laboratory of Biology and Genetic Improvement of Oil Crops, Ministry of Agriculture, Oil Crops Research Institute of the Chinese Academy of Agricultural Sciences (CAAS), Wuhan 430062, China; b National Nanfan Research Institute (Sanya), Chinese Academy of Agricultural Sciences, Sanya 572024, China; c Department of Horticulture, College of Agriculture and Natural Resources, Debre Markos University, Debre Markos, P.O. Box. 269, Amhara, Ethiopia

**Keywords:** Climate adaptation, Root adaptation, Root architecture, Root-soil interactions

## Abstract

Peanut (*Arachis hypogaea* L.) cultivation is increasingly vulnerable to climate change, with drought and heat stress emerging as major constraints to productivity and food security. This review explores the critical role of root architecture in enhancing peanut adaptation to environmental stressors, and evaluates current strategies and future directions for improving root traits through genetic, physiological, and agronomic approaches. Efficient root systems, characterized by deeper rooting and optimized xylem design, significantly improve water and nutrient acquisition under drought conditions. Key regulators such as abscisic acid (ABA), strigolactones, and specific root-related genes modulate root development and stress responses. Root exudates further enhance soil root interactions, while the peanut root microbiome contributes to nutrient cycling and resilience. Biotechnological tools, including quantitative trait loci (QTL) mapping and CRISPR/Cas-based genome editing, are being harnessed to manipulate root traits at the molecular level. Agronomic practices like mulching and cover cropping synergize with genetic improvements by enhancing soil structure and moisture retention. Strengthening peanut root architecture through the integration of modern breeding, biotechnological advances, and sustainable soil management offers a promising path toward climate-resilient peanut production. Future research should prioritize the convergence of these approaches, alongside microbiome exploration, to secure yield stability and food security in a changing climate.

## Graphical abstract

**Figure:**
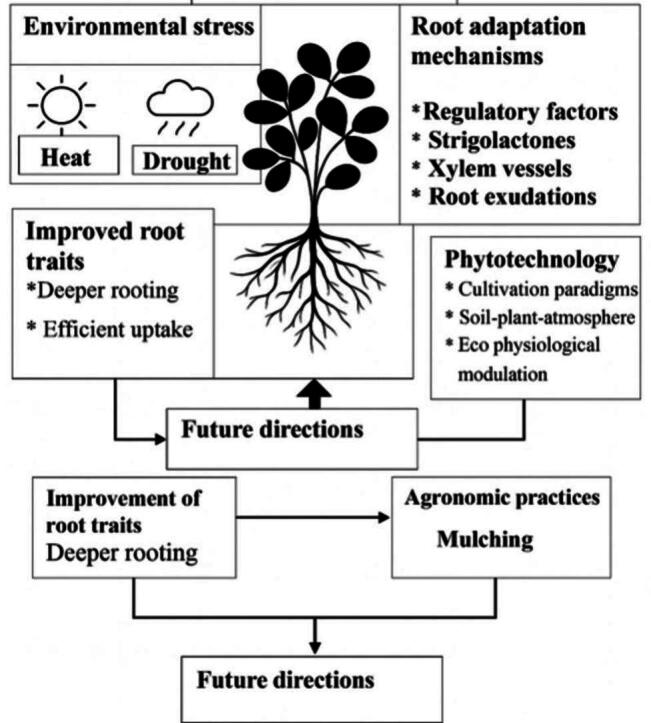

## 1 Introduction

Peanut (*Arachis hypogaea* L.) is a leguminous crop of significant economic and nutritional importance, cultivated widely across tropical and subtropical regions.^1^ Peanut's ability to thrive in diverse environments has made it an essential staple, providing nourishment to millions of people around the world.^2^ However, the growing challenges of climate change including rising temperatures, more frequent droughts, and unpredictable rainfall are increasingly threatening peanut production. Understanding the crop’s physiological mechanisms, particularly its root architecture, is essential for developing strategies to strengthen its resilience against climate-related stresses. The structure of a plant’s root system is vital for efficiently absorbing water and nutrients, particularly under challenging environmental conditions.^3^ In peanuts, the root system consisting of a deep taproot and lateral roots allows the plant to reach deeper soil layers, ensuring access to water during drought conditions. Research on peanut root response to drought stress suggests that while root growth may slow down temporarily during early drought conditions, it tends to recover once water availability is restored.^4^ Root plasticity the ability of roots to adapt and grow in response to changing environmental conditions plays a key role in enhancing the crop's overall adaptability. Correspondingly, research published in the Journal of Plant Physiology on root architecture and plant productivity states that, as peanut roots grow underground, they are the first to detect changes in the external environment. Thus, they adjust their genetic programming for post-embryonic development to ensure survival.^5^ Accordingly, to cope with climate change, peanuts use various root-based strategies, including changes in root-to-shoot signaling, enhanced water uptake efficiency, and adjustments in root-soil interactions, such as forming beneficial partnerships with mycorrhizal fungi.

The rhizosphere microbiome and root exudates play a crucial, yet often underexplored, role in enhancing peanut resilience to climate stress. Root exudates comprising sugars, amino acids, organic acids, and secondary metabolites act as chemical signals and energy sources that actively shape the microbial community surrounding the roots.^6^, ^7^ In peanuts, these exudates facilitate symbiotic interactions with beneficial soil microbes, including nitrogen-fixing rhizobia and arbuscular mycorrhizal fungi, which enhance nutrient acquisition and improve plant tolerance to drought and heat.^8^ These microbial partners can modulate hormone signaling, boost antioxidant activity, and improve water-use efficiency, forming a dynamic feedback system between the plant and its environment. As climate variability intensifies, harnessing and optimizing these root-microbe interactions offers a promising strategy to develop climate-resilient peanut varieties.^9^, ^10^ Integrating microbiome-focused approaches with root phenotyping and breeding could significantly advance sustainable agriculture and crop adaptability under stress-prone conditions.^11^, ^12^

Despite the recognized importance of root architecture in adapting to climate change, several challenges impede progress in this area. One of the main obstacles is the limited focus on understanding the genetic, molecular, and physiological mechanisms that govern root development and function under stressful conditions. A review article on sustaining peanut yield and nutritional quality in harsh environments reports the availability of advanced breeding tools to improve both productivity and nutritional value. It also suggests that a holistic breeding approach, which includes traits for drought and heat tolerance, could be an effective strategy for developing climate-resilient peanut varieties with enhanced nutritional quality.^13^ Furthermore, the complexity of root-soil interactions, especially the symbiotic relationships with soil microbiota like mycorrhizal fungi, is still not fully understood. Additionally, the challenge of phenotyping root traits due to their underground nature makes traditional breeding methods time-consuming and labor-intensive. Thus, to address these limitations, there is a need for advanced genomic tools, integrated research approaches, and innovative breeding techniques to develop peanut varieties that can withstand the diverse challenges posed by climate change.^14^ Similarly, a review paper on advances in *Arachis* genomics for peanut improvement states that the lack of adequate genomic resources has significantly hindered molecular breeding efforts, leaving peanuts as one of the less-studied crops.^15^ Thus, this work aims to review how the root architecture of peanut contributes to climate adaptation, promoting sustainable agriculture and enhancing crop resilience.

## 2 Root system architecture of peanut crop

The root system architecture of peanuts is essential to their growth and resilience, especially when faced with challenging environmental conditions.^16^ Research on the differential physio-biochemical and metabolic responses of peanuts under various abiotic stress conditions suggests that superior peanut cultivars with enhanced stress tolerance can be developed by combining identified molecular phenotypes with traditional breeding or genetic engineering techniques.^17^ Peanuts are also known for their prominent taproot, which extends deep into the soil, supported by a widespread network of lateral roots. This deep taproot allows the plant to access water and nutrients from lower soil layers, which is crucial during drought conditions. Also, a critical review on the physiological responses of groundnuts to drought stress and its mitigation reports that applying knowledge systematically can lead to significant improvements in both yield and yield stability in global groundnut production.^18^ In addition, lateral roots that spread horizontally play a vital role in absorbing nutrients near the soil surface while also providing structural support. For example, a study on the growth and physiology of groundnuts proves that an extensive root system, combined with the ability to extract moisture during periods of soil moisture deficit, can help delay dehydration and extend the productive period of the plant.^19^ As a result, the deep and widespread root systems enable it to thrive in different soil types, enhancing its resilience to water and nutrient shortages.

Moreover, the peanut root system reveals remarkable adaptability, enabling the plant to modify its root growth in response to changing environmental conditions, including soil moisture levels and nutrient availability.^20^, ^21^ A study on the physiological plasticity of 60 *Arachis hypogaea* cultivars under natural drought conditions in India's semi-arid regions reports that plasticity is a vital adaptive mechanism, and it allows the crop to survive and thrive in areas with inconsistent rainfall and unpredictable climate patterns.^22^ Furthermore, the root system forms symbiotic relationships with soil microbiota, particularly mycorrhizal fungi, which play a crucial role in enhancing nutrient absorption and strengthening the plant’s resilience to environmental stress. For instance, a study on the physiological mechanisms behind *Phomopsis liquidambari*-mediated symbiosis in a monocropping system found that improved symbiotic efficiency in continuously cultivated peanuts was primarily associated with enhanced plant nutrition rather than the activation of plant defense mechanisms.^23^ The structural complexity and adaptability of the peanut root system play a vital role in helping the plant endure the diverse challenges brought on by climate change. Generally, the major components of the peanut root architecture include a deep taproot that provides stability and access to moisture, along with an extensive network of lateral roots that facilitate nutrient and water absorption, adventitious and nodulated roots contribute to overall plant support and improve nitrogen fixation, further enhancing the crop’s resilience (Table 1).

**Table 1:** The root system structures of the peanut crop.

No.	Root systems	Structures for climate adaptation	Ref.
1	Adventitious roots	Roots that offer extra support and absorb nutrients.	^24^
2	Fibrous roots	Thin, hair-like roots densely surround the primary and lateral roots, enhancing the plant’s ability to absorb water and nutrients from the upper soil layers.	^25^
3	Lateral roots	Roots branching from the taproot absorb nutrients and water from the soil.	^26^
4	Taproot	A central, thick root that anchors the plant and absorbs deep water.	20, 27
5	Primary root	The first root from the seed, which grows into the taproot.	^28^
6	Secondary roots	Roots emerging from the primary root and branching into tertiary roots expand the surface area, improving nutrient absorption efficiency.	^29^
7	Nodulated roots	Peanut roots form symbiotic nodules with nitrogen-fixing bacteria (*Rhizobia*), essential for nitrogen assimilation.	30, 31

## 3 Impact of climate change on peanut growth

**Fig. 1:**
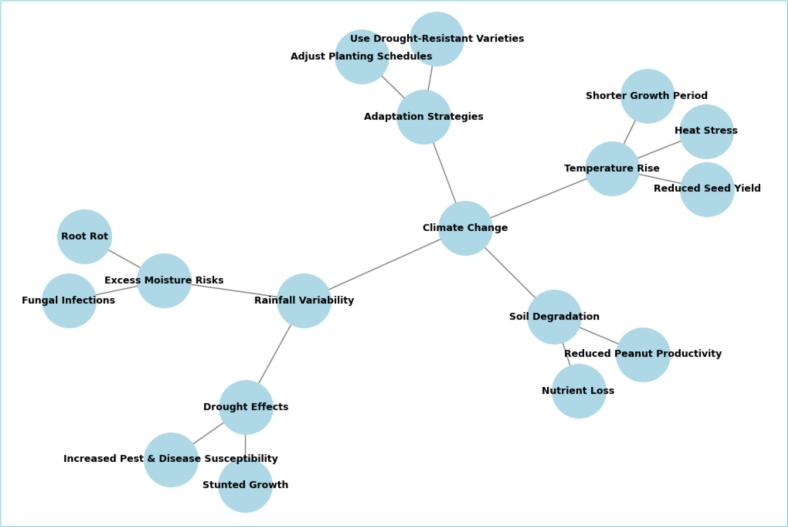
Climate change influences peanut growth.

Climate change has a profound impact on peanut growth by altering the environmental conditions necessary for its development. A study examining the effects of a 2 °C temperature rise on peanut production in Guilan Province, Iran, revealed that the crop’s growth period was reduced from 142 to 123 days. However, despite the shorter growth cycle, the yield increased by 8.73 %.^32^ Rising temperatures can cause heat stress, which negatively affects main growth stages such as flowering and pod development in peanuts. Excessive heat can also hinder seed formation, ultimately leading to lower yields. Research on the effects of high temperatures on peanut reproduction and yield under both ambient and elevated carbon dioxide levels indicates that increased CO_2_ does not counteract the adverse impacts of heat. The findings suggest that future climate conditions, particularly in regions where current temperatures are already near or above optimal levels, will likely result in reduced peanut seed yields.^33^ Changes in rainfall patterns, whether leading to drought or excessive moisture, can adversely affect peanut growth. Inconsistent water availability can stunt plant development, weaken resistance to pests and diseases, increase the risk of root rot, and create favorable conditions for fungal infections (Fig. 1). These challenges ultimately compromise the overall health and productivity of the crop.^34^

Furthermore, climate change leads to soil degradation and alters nutrient availability, creating additional challenges for peanut cultivation (Fig. 1). Research from the AgMIP Coordinated Climate-Crop Modeling Project (C3MP) indicates that focusing only on seasonal changes in temperature and precipitation misses the impact of sub-seasonal variability, which can significantly affect peanut yields in different regions.^35^ Thus, farmers especially in developing countries like Asian, and African nations such as India, Nigeria, Sudan, and Senegal, may need to modify their farming practices. This could involve adjusting planting schedules and using drought-resistant crop varieties to better cope with the changing climate.

## 4 Mechanisms of root adaptation to drought stress

Peanut plants have developed various root adaptation mechanisms to withstand drought stress, helping them survive and maintain productivity in water-scarce environments. For instance, a book chapter on “Groundnut: Genetic approaches to enhance adaptation to drought” indicate that current research on improving groundnut crops for drought resilience has identified a few promising strategies to address these challenges.^36^, ^37^ One main adaptation is the modification of root architecture, where peanuts develop deeper and more extensive root systems in response to limited soil moisture. This adaptation enables the plants to tap into water reserves from deeper soil layers, which is especially crucial during extended drought periods.^16^ Peanuts can also adapt by increasing the number of lateral roots, allowing them to explore a larger soil volume for water and nutrients. Over time, peanut plants adjust to drought by growing deeper roots, expanding lateral root networks, and boosting aquaporin activity to absorb water more efficiently. However, a study on the response of root growth and development to nitrogen and potassium deficiencies, as well as microRNA-mediated mechanisms in peanuts, found that a lack of these essential nutrients significantly reduces root length, surface area, volume, and vitality.^13^ In addition, root respiration weakens under nutrient deficiency, and peanut roots respond differently to nitrogen and potassium stress, possibly due to microRNA-regulated pathways and mechanisms.^13^ Hence, by expanding their root surface area, peanut plants enhance their ability to absorb water and essential nutrients, increasing their resilience in drought conditions.

Furthermore, peanut roots have developed multiple adaptive mechanisms to improve drought resilience, including the regulation of aquaporins specialized proteins that control water movement across cell membranes. Research on the physiological, anatomical, transcriptomic, and metabolomic responses of two distinct peanut cultivars under acute drought stress at the seedling stage found that drought-tolerant varieties utilize an advanced terpene skeleton synthesis pathway, supplying essential precursors for both primary and secondary metabolism.^38^ Under drought stress, peanuts can enhance the expression of specific aquaporins to improve water uptake efficiency. Furthermore, they produce osmolytes like proline, which help maintain cellular turgor and protect root cells from osmotic stress, ensuring better resilience in water-limited conditions.

Research on the stress-inducible overexpression of AtHDG11 in three independent homozygous transgenic peanut lines has shown significant improvements in drought and salt tolerance. This enhancement was achieved through the upregulation of stress-responsive genes, including those involved in aquaporin expression, antioxidative enzyme activity, and free proline accumulation, all of which contribute to the plant’s ability to withstand environmental stress.^39^ These physiological adaptations allow peanut plants to sustain root function and overall growth, even in water-limited conditions, enhancing their ability to survive and thrive despite environmental challenges.

Moreover, the relationship between peanut roots and soil microorganisms is crucial for enhancing the plant’s ability to withstand drought stress.^40^, ^41^ Peanut plants release root metabolites that attract beneficial soil microbes, such as mycorrhizal fungi and certain bacteria. A study on the interaction between groundnut roots and *Pseudomonas sp*. found that root colonization by RP2 triggered the exudation of metabolites that facilitated microbial attachment, suppressed fungal growth, promoted plant growth, and enhanced the expression of defense-related proteins in the roots.^42^ Mycorrhizal associations play a crucial role in improving nutrient and water uptake by expanding the root surface area and enhancing access to soil moisture. Correspondingly, certain beneficial bacteria support plant growth and stress resilience by producing phytohormones that stimulate root development, helping peanuts better withstand drought conditions.^43^, ^44^ As a result, these beneficial interactions between peanut roots and soil microorganisms play a vital role in the plant's overall resilience and adaptability to drought stress, helping ensure successful growth even in challenging environments.

Genes and their possible mechanisms involved in peanut root adaptation to climate changes represent a complex regulatory framework that enables the plant to maintain growth and function under stress.^45^, ^46^ Various genes are involved in sensing and responding to environmental cues, including temperature fluctuations, water scarcity, and soil salinity (Table 2). These genes regulate crucial processes such as root elongation, branching, and suberization, which collectively enhance the plant’s ability to access water and nutrients under unfavorable conditions. Gene expression changes are often mediated through stress-responsive signaling pathways that involve transcription factors, protein kinases, and hormone biosynthesis regulators, allowing the root system to adapt both structurally and functionally.^47^

**Table 2:** Genes and their possible mechanisms involved in peanut root adaptation to climate changes.

Genes	Possible mechanism of adaptation	Ref.
AhDREB1	Involved in drought and cold stress tolerance through ABA-independent pathway.	^51^
AhNAC2	Regulates root architecture and stress-responsive genes under drought stress.	^52^
AhWRKY75	Enhances root elongation and modulates oxidative stress under abiotic conditions.	^53^
AhLEA3	Protects cells from dehydration by stabilizing proteins and membranes.	^54^
AhHSP70	Provides protection against heat stress by preventing protein aggregation.	^55^
AhCBF1	Enhances cold tolerance by regulating cold-responsive (COR) genes.	^56^
AhNCED1	Involved in ABA biosynthesis, critical for root adaptation to drought.	57, 58
AhP5CS	Facilitates proline accumulation, enhancing osmotic adjustment during stress.	^59^
AhMYB44	Modulates the expression of root stress-responsive genes under salt and drought conditions.	60, 61
AhGSTU4	Participates in detoxification and oxidative stress response in roots.	^62^

Moreover, gene networks associated with oxidative stress response, cell wall modification, and osmotic balance contribute to improved root resilience.^48^ These mechanisms include the upregulation of protective enzymes, accumulation of compatible solutes, and activation of transporters that facilitate ion and nutrient uptake.^49^ Many of these genes exhibit coordinated expression patterns, suggesting a highly integrated response to climate stress.^50^ Consequently, future research focusing on functional validation and the integration of gene expression data with physiological traits will be key to identifying candidate genes for breeding climate-resilient peanut varieties with optimized root architecture and enhanced stress tolerance.

Drought stress causes osmotic imbalance and reactive oxygen species accumulation, initiating abscisic acid (ABA) signalling.^63^ AhNCED1 enhances ABA biosynthesis, while AhNAC2 and AhDREB1 act as key transcription factors regulating stress-responsive genes. Together, these genes coordinate protective mechanisms such as stomatal closure, osmolyte accumulation, and antioxidant defense, leading to improved drought tolerance and plant adaptation (Fig. 2a).

**Fig. 2a:**
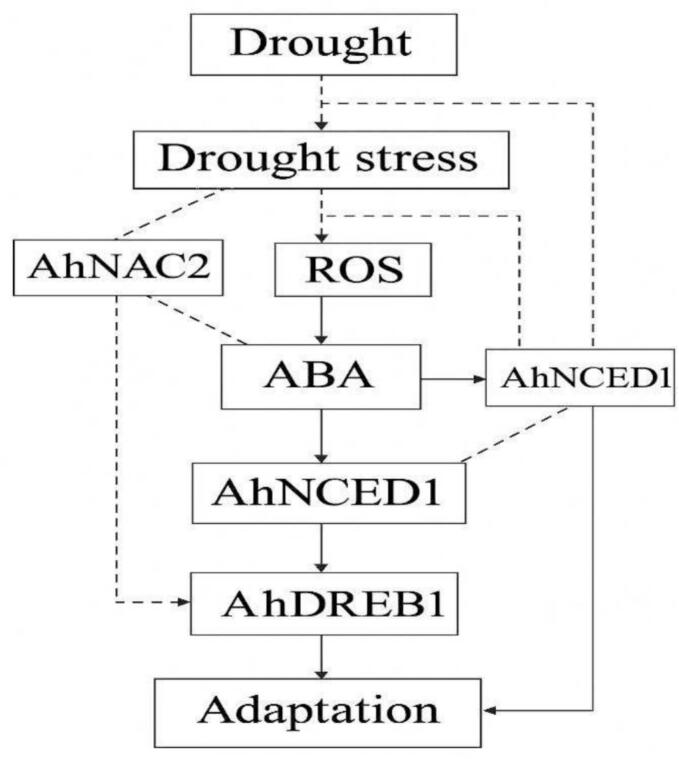
Drought stress adaptation pathway.

Salinity stress triggers osmotic imbalance and oxidative stress, leading to the activation of stress-responsive pathways.^64^ AhMYB44 regulates ABA signaling, enhancing stress tolerance, while AhP5CS promotes proline biosynthesis for osmotic adjustment.^65^ These coordinated responses help the plant achieve adaptation under high salinity conditions (Fig. 2b).

**Fig. 2b:**
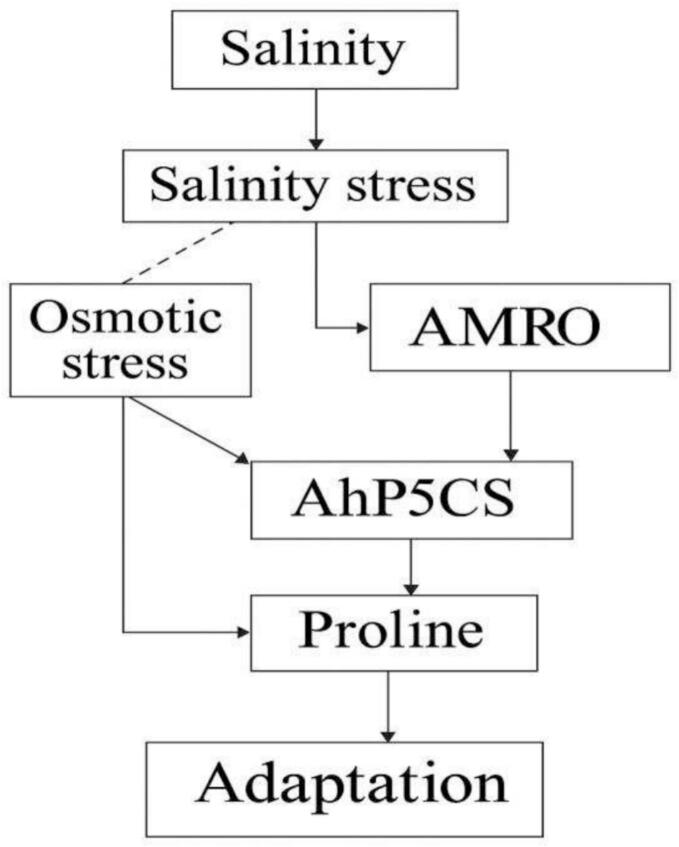
Salinity stress adaptation pathway.

The plant's adaptive response to cold stress begins with the onset of cold stress, activating AhCBF1.^66^ This gene triggers key cellular processes such as signal transduction and reactive oxygen species (ROS) homeostasis. Subsequently, AhCBF1 activates AhDREB1, which regulates mechanisms like osmotic adjustment and antioxidant protection, ultimately leading to plant adaptation.^67^ The diagram clearly outlines the interrelationships among these processes, providing a detailed pathway for the plant’s response to cold stress (Fig. 2c).

**Fig. 2c:**
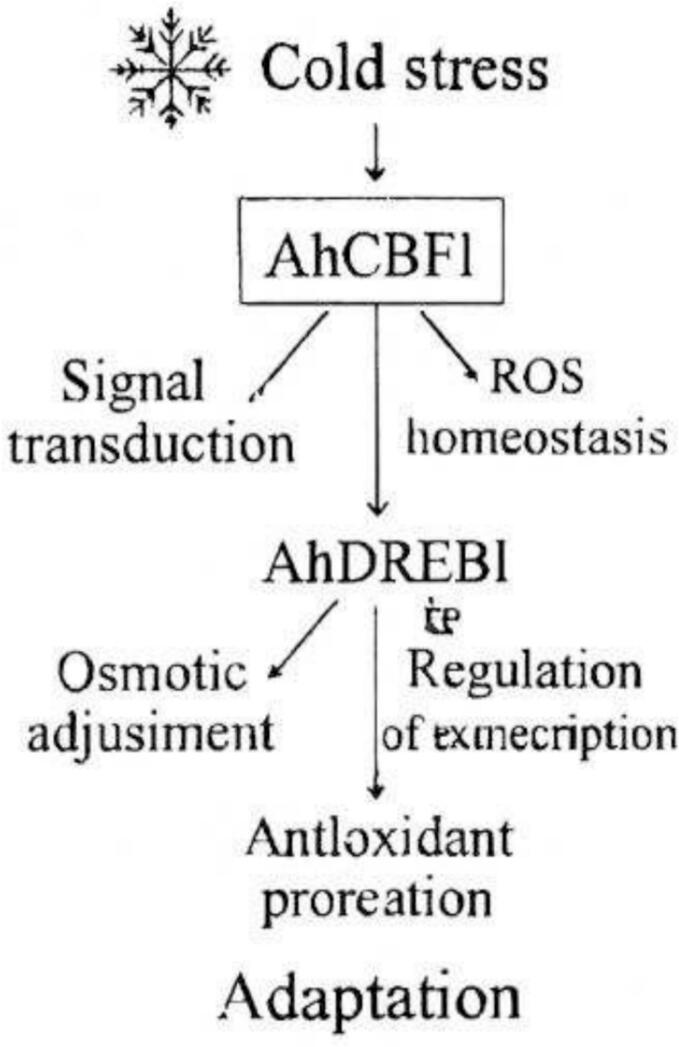
Cold stress adaptation pathway in plants.

Heat stress activates AhHSP70, triggering protein folding and ROS scavenging.^68^ Concurrently, AhWRKY75 regulates osmotic balance, gene expression, and antioxidant defense mechanisms.^68^ These processes work together to enable the plant's adaptation to heat stress (Fig. 2d).

**Fig. 2d:**
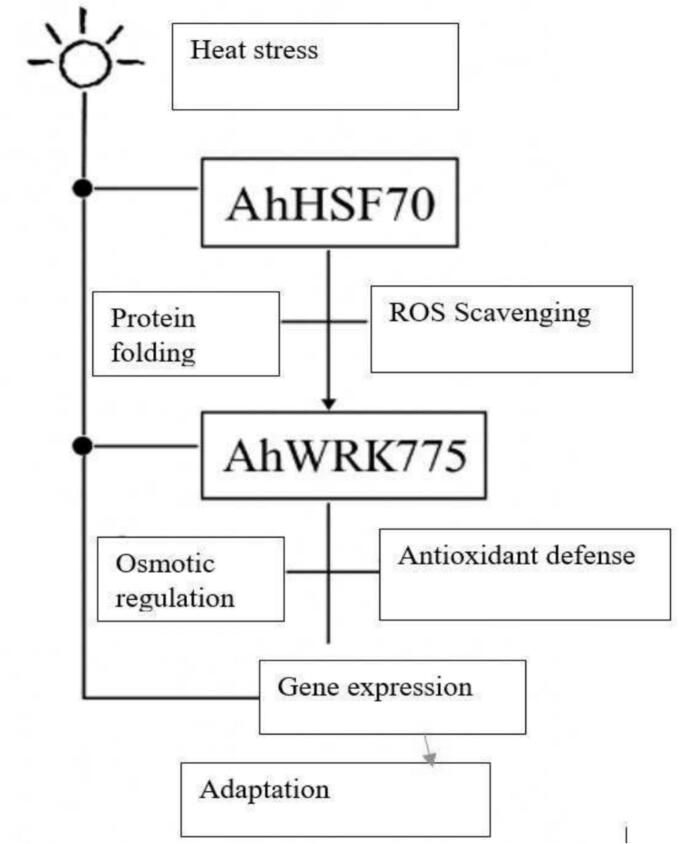
Heat stress adaptation pathway in plants.

## 5 Nutrient uptake and soil interaction under climate stress

**Fig. 3:**
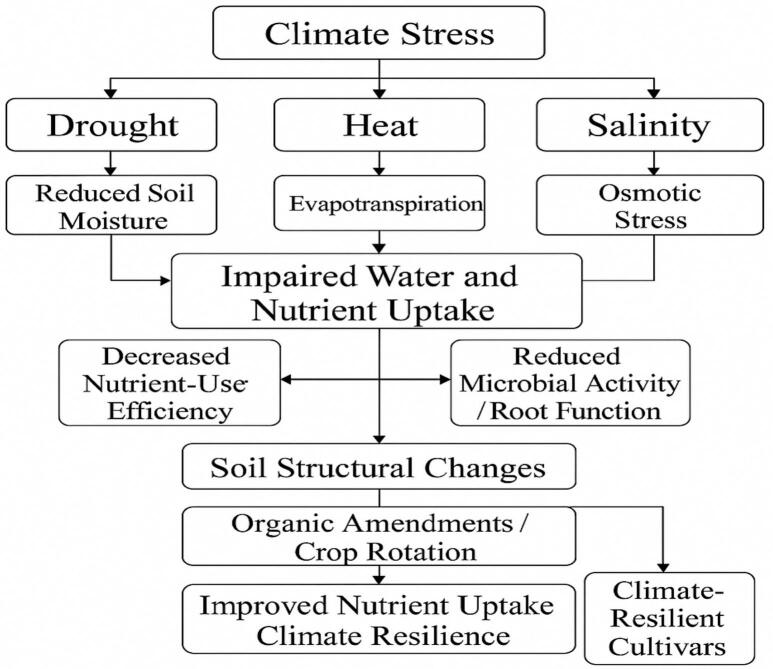
Impact of climate stress on peanut nutrient uptake and soil health.

Peanut crops encounter substantial difficulties in nutrient uptake when faced with climate-related stresses such as drought, heat, and rising salinity. A book chapter on climate-smart groundnuts for achieving high productivity and improved quality: current status, challenges, and opportunities notes that increasing CO_2_ levels, unpredictable rainfall, fluctuating humidity, brief periods of high temperatures, and salinity all negatively affect the plant’s physiology, disease resistance, fertility, yield, and seed nutrient content.^69^ Hence, climate stress can alter the physical and chemical properties of the soil, affecting the availability and absorption of nutrients by plants (Fig. 3). For instance, prolonged droughts decrease soil moisture, which in turn limits the movement of essential nutrients such as nitrogen, potassium, and phosphorus important elements necessary for peanut growth.^34^ Heat stress also increases evapotranspiration, which further dries out the soil and can lead to nutrient deficiencies.^70^, ^71^ The relationship between soil and peanut crops under stress is complex. Stressed soils often experience reduced microbial activity, which is crucial for nutrient cycling. In addition, rising temperatures and unpredictable rainfall intensify salinity, which can impair the crop's ability to absorb nutrients by affecting root function and soil structure. A study on peanut production in the saline-alkali lands of the Yellow River Delta reports that soil salinity is a major factor impacting pod development and yield, and optimizing planting methods in these areas is essential for improving peanut production.^72^ Salt accumulation in the soil induces osmotic stress, thereby impairing the ability of peanut plants to efficiently absorb water and nutrients. For instance, a study on the impact of salinity stress on the growth, seedling development, and water consumption of peanut (*Arachis hypogaea* cv. NC-7) found that while saline water with a concentration of less than 4 dS m^−1^ had positive effects on plant growth and development, water with a salinity above 4 dS m^−1^ negatively affected these same parameters.^73^ Overall, higher salinity levels led to an increase in sodium concentration in the leaves and roots (Fig. 3). Consequently, nutrient-use efficiency in peanut crops declines, indicating that even when nutrients are present in the soil, the plants exhibit reduced capacity for effective absorption and utilization. To mitigate this challenge, it is imperative to adopt sustainable soil management practices such as the application of organic amendments and implementation of crop rotation alongside the development and deployment of climate-resilient peanut cultivars. These integrated strategies are critical for enhancing nutrient uptake and alleviating the adverse impacts of climate change on crop productivity.

The regulated and interacted genes involved in nutrient uptake and soil response under climate stress in peanut are integral to the plant’s adaptive capacity and overall resilience (Table 3). Under stress conditions such as drought, salinity, and nutrient deficiency, specific genes are activated to regulate the transport, assimilation, and redistribution of essential nutrients.^74^, ^75^ These genes work in coordination with signaling pathways and hormonal responses to maintain nutrient homeostasis and support root plasticity.^76^ In addition, interactions between root-expressed genes and the rhizosphere environment help to optimize nutrient acquisition and soil adaptation.^77^ Thus, the regulation of these genes is dynamic and often involves complex cross talk between various molecular networks, enabling the peanut plant to adjust to changing environmental conditions.

**Table 3:** Regulated and interacted genes of nutrient uptake and soil under climate stress in peanut.

**Gene family**	**Functions and regulations**	Ref.
AhNRT1	Nitrate transporter gene, upregulated under drought stress.	^78^
AhPHT1	Phosphate transporter gene, involved in Pi uptake.	^79^
AhZIP1	Zinc transporter, responsive to low Zn conditions.	^80^
AhAMT1-1	Ammonium transporter gene, induced under salt stress.	^81^
AhFER	Iron uptake regulation gene, modulated by root-soil interaction.	^82^
AhHAK5	Potassium transporter activated during nutrient deficiency.	^83^
AhNRAMP	Metal ion transporter for Mn and Fe under climate variability.	84, 85

Under drought stress, limited water availability reduces nutrient solubility and mobility in the soil, impairing plant uptake.^86^ This triggers abscisic acid (ABA) signaling, activating drought-responsive genes such as DREB2A/B and NCED3, which regulate root architecture and stress adaptation. The expression of key nutrient transporters like PHT1;4 (phosphate) and NRT1.1 (nitrate) is modulated to enhance efficiency under stress. As roots grow deeper and thinner, the plant shifts from active to more passive nutrient uptake strategies to survive in low-water conditions (Fig. 4a).

**Fig. 4a:**
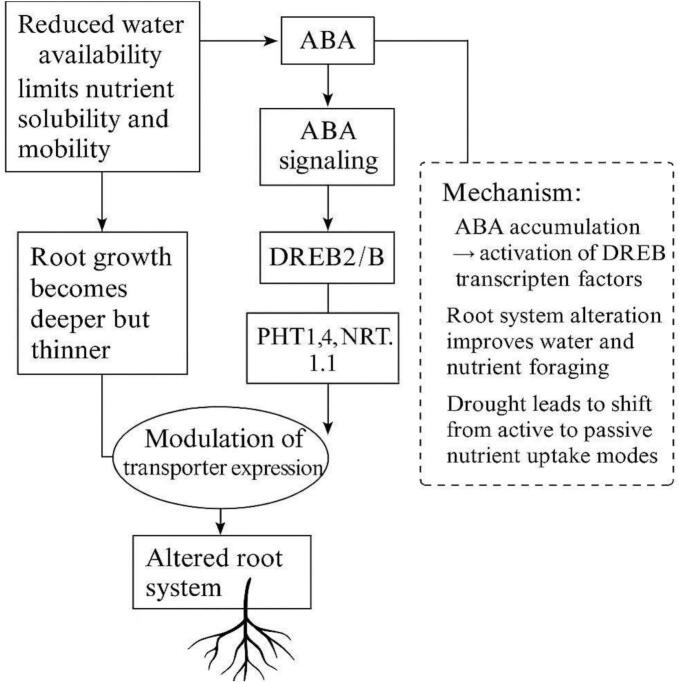
Drought stress pathway and nutrient uptake regulation.

Salinity stress disrupts ion homeostasis, mainly through excessive Na^+^, which inhibits K^+^ and Ca^2+^ uptake, leading to osmotic and ionic stress.^87^ The SOS pathway (SOS1/2/3) and transporters like NHX1 and HKT1 are activated to expel or compartmentalize sodium, preserving cytosolic balance.^88^, ^89^ HAK5 upregulation ensures continued potassium acquisition.^90^ Root function and nutrient transport are supported by H^+^-ATPase activity,^91^ which energizes ion transport across membranes, enabling plants to sustain nutrient uptake in saline environments (Fig. 4b).

**Fig. 4b:**
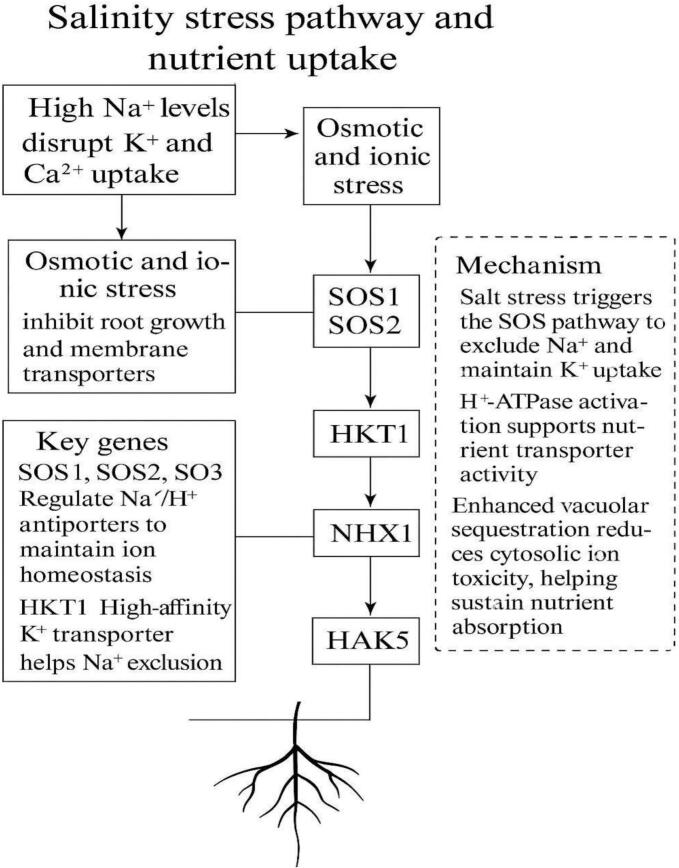
Salinity stress response and ion homeostasis mechanisms.

Cold stress reduces membrane fluidity and slows root growth, limiting nutrient uptake.^92^ Transporter activity declines, especially for nitrate and potassium, and genes like CBF1/2/3, NRT2.1, and CIPK23 mediate cold responses and nutrient transport.^93^, ^94^ Calcium signaling and the CBF pathway enhance cold protection by stabilizing transporter function (Fig. 4c).

**Fig. 4c:**
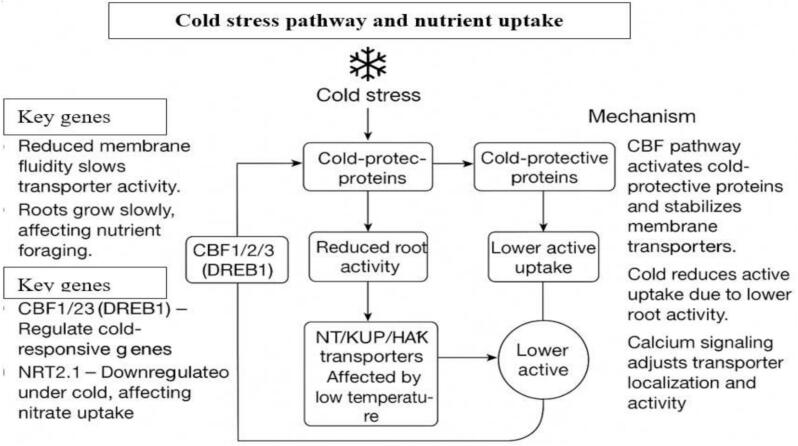
Cold stress pathway and nutrient uptake.

Heat stress disrupts membrane integrity and enzyme activity, impairing nutrient transport.^95^ Key genes like HSFA1/HSFA2 and HSPs are activated to stabilize proteins and transporters.^96^, ^97^ Increased transpiration can temporarily enhance nutrient flow but may lead to imbalances. ROS scavenging helps to protect root integrity under heat conditions (Fig. 4d).

**Fig. 4d:**
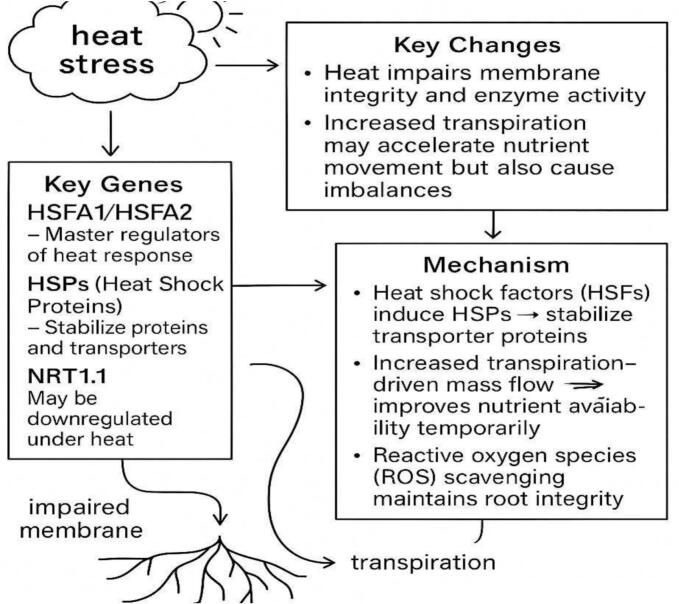
Heat stress pathway and nutrient uptake.

## 6 Genetic and molecular basis of root adaptation

The genetic and molecular foundations of root adaptation in peanut crops are crucial for enhancing their resilience to environmental stresses, especially drought and poor soil conditions. Key genes involved in root architecture and function play a vital role in improving water and nutrient uptake, ultimately boosting the crop's ability to thrive in challenging environments.^98^ An invited review on the challenges of designing root system architecture (RSA) adapted to climate change emphasizes that roots are crucial for absorbing water and nutrients. The architecture of the root system determines the soil area from which these resources can be accessed.^99^ A doctoral dissertation on leveraging genomic mapping and QTL analysis to improve drought tolerance in cultivated peanuts reveals that certain genes regulate root length, density, and branching patterns, which enhance water acquisition during drought conditions.^100^ By improving root traits, such as developing deeper root systems, peanut crops can access moisture and nutrients from deeper layers of soil, boosting their resilience to stress. At the molecular level, signaling pathways involving hormones and auxins play an important role in root development and stress responses in peanuts. For example, research on differential gene expression and pathways in response to severe drought stress in peanuts describe that numerous genes are involved in regulating these networks under drought conditions.^101^ Besides, stress-responsive genes, such as those involved in ROS detoxification and osmotic adjustment, help peanut roots adapt to adverse conditions.^102^ Recent advances in genomics, transcriptomics, and CRISPR gene-editing technologies have opened up new possibilities for modifying molecular pathways. These advancements make it possible to develop peanut varieties with root systems that are better equipped to handle environmental stress, ensuring sustainable yields even in the face of a changing climate.

In general, the pathways involved in root adaptation to climate changes in peanut encompass a complex network of molecular, physiological, and biochemical processes that enable the plant to cope with environmental stressors (Table 4). The key pathways include hormone signaling, antioxidant defense systems, osmolytes biosynthesis, and cell wall remodelling.^103^ These pathways regulate root growth direction, depth, and architecture, facilitating improved water and nutrient acquisition under stress conditions like drought and salinity.^104^, ^105^ Moreover, crosstalk among signaling pathways and transcriptional regulatory networks significantly enhances the plant’s ability to adapt to fluctuating environmental conditions. Therefore, elucidating these mechanisms is crucial for identifying genetic targets that can be used in breeding programs to improve climate resilience in peanut crops.

**Table 4:** The pathways involved in root adaptation to climate changes in peanut.

S/No.	Pathway	Ref.
1	Hormonal signaling (e.g., ABA, auxin, cytokinin).	^103^
2	Root architectural changes (e.g., deeper rooting).	^106^
3	Gene expression and transcriptional regulation.	^107^
4	ROS signaling.	^108^
5	Epigenetic modifications.	^109^
6	Aquaporin regulation.	^110^
7	Symbiotic interactions (e.g., mycorrhizae).	^111^
8	Cell wall remodeling.	103, 112

Recent transcriptomic and metabolomic studies have significantly advanced the understanding of root adaptation mechanisms in various crops, including peanut, under climate-induced stresses such as drought, heat, and salinity (Table 5). Transcriptomic analyses have identified key stress-responsive genes and regulatory networks involved in root architecture modulation, hormonal signaling, and nutrient uptake.^113^, ^114^ Concurrently, metabolomic investigations have characterized vital biochemical compounds, including flavonoids, amino acids, and organic acids, which contribute to cellular protection and metabolic adjustments during abiotic stress.^115^, ^116^ Collectively, these approaches provide a comprehensive framework for elucidating complex molecular responses in peanut root systems under adverse environmental conditions.

**Table 5:** Transcriptome and metabolomics studies in root adaptation to climate changes in peanut.

S/No	Study type	Ref.
1	Transcriptome analysis of drought-tolerant vs. susceptible peanut varieties.	^118^
2	Differential gene expression in roots under heat stress.	^101^
3	RNA-seq analysis of root development under salinity.	^119^
4	Metabolite profiling during root adaptation to low phosphorus.	^38^
5	Combined transcriptome and metabolomics under water-deficit.	^120^
6	Identification of key pathways (e.g., flavonoid biosynthesis).	121, 122
7	Root exudate composition under stress via metabolomics.	^123^

Despite significant progress, gaps remain in translating omics insights into practical breeding applications. Thus, future research should prioritize the integration of transcriptomic and metabolomic datasets with phenotypic and agronomic traits through advanced computational modeling and multi-omics platforms. High-resolution spatial and temporal profiling, coupled with genome-wide association studies (GWAS) and gene-editing technologies, will be crucial in identifying functional markers and regulatory hubs.^117^ Hence, such integrative strategies are essential for the development of climate-resilient peanut cultivars with enhanced root plasticity and improved resource-use efficiency.

## 7 Root-based management practices for climate resilience

Root-based management practices are crucial for improving the climate resilience of peanut crops, particularly in areas affected by drought, heat stress, and poor soil fertility. A study on the effects of soil tillage, management practices, and mulching film application on soil health and peanut yield in continuous cropping systems suggests that using green manure with mulching film is an effective strategy.^124^ This approach helps to improve soil physicochemical and microbial properties while also boosting peanut pod yield, even when considering the residual pollution caused by plastic films. One of the most effective ways to enhance peanut growth is by optimizing soil health through practices like cover cropping and organic mulching. These methods improve soil structure, boost moisture retention, and increase microbial activity, all of which directly support root development and function.^125^ For example, it's been reported that cover crops help reduce soil erosion and improve organic matter, allowing peanut roots to grow deeper and stronger in search of water and nutrients.^126^ Moreover, mulching helps conserve soil moisture by reducing evaporation, which in turn mitigates the effects of heat stress on the root systems.^127^ Maintaining a healthy root zone that can efficiently absorb nutrients during extreme conditions is crucial for the overall resilience of the crop.

Previous studies have also reported important root-based management practices, such as deep tillage and subsoiling, which help break up compacted soil layers, allowing peanut roots to grow deeper.^128^ This practice helps peanuts access moisture reserves deeper in the soil, which is crucial during droughts. A study on simulating drought tolerance in peanut varieties found that breeders are focusing on selecting high-yielding genotypes that thrive in water-limited environments. Recently, several peanut varieties with enhanced drought tolerance have been developed.^129^ In addition to physical methods, crop rotation with legumes can enhance soil nitrogen levels, which supports healthier root systems. A study on rotational strip intercropping of maize and peanuts found that this practice boosted productivity by improving photosynthetic production, soil nutrients, and bacterial communities. Over a six-year period, the land equivalent ratio (LER) for this rotation averaged 1.19, demonstrating its effectiveness.^130^ Incorporating legumes into crop rotations, helps boost nitrogen fixation, which reduces the need for fertilizers and improves nutrient availability. Root-based strategies also play a crucial role in strengthening peanut crops against climate stress. Overall, peanuts adapt to climate change through mechanisms like deep root systems that access moisture during droughts and improved water-use efficiency. Additionally, they can adjust their flowering times and delay leaf aging, which helps them, had better withstand extreme weather conditions (Table 6).

**Table 6:** Adaptation mechanisms of peanut crops to climate change (summary).

No.	Climate change adaptation mechanisms		Ref.
1	Deep root system	The ability of peanuts to develop a taproot that reaches deeper soil moisture during droughts.	^131^
2	Water use efficiency	Adaptations that improve water efficiency, such as regulating stomatal closure to minimize water loss.	^132^
3	Drought tolerance genes	The expression of specific genes that help the plant respond to drought stress, including those involved in osmotic adjustment.	^118^
4	Heat stress resilience	Mechanisms that enhance heat tolerance by increasing the activity of antioxidant enzymes, helping to protect the plant from heat damage.	^133^
5	Symbiotic nitrogen fixation	Nodule formation with Rhizobia bacteria improves nitrogen availability, supporting growth in nutrient-poor conditions.	^134^
6	Reduced leaf area	A strategy to minimize water loss by reducing leaf surface area during periods of heat and drought.	135, 136
7	Delayed senescence	The ability to delay leaf aging, which extends photosynthesis and strengthens resilience under challenging conditions.	^137^
8	Flexible flowering and maturity timing	Adjusting the timing of flowering and maturity to avoid extreme weather conditions, such as drought or heat.	138, 139

## 8 Future directions in peanut research and climate adaptation

**Fig. 5:**
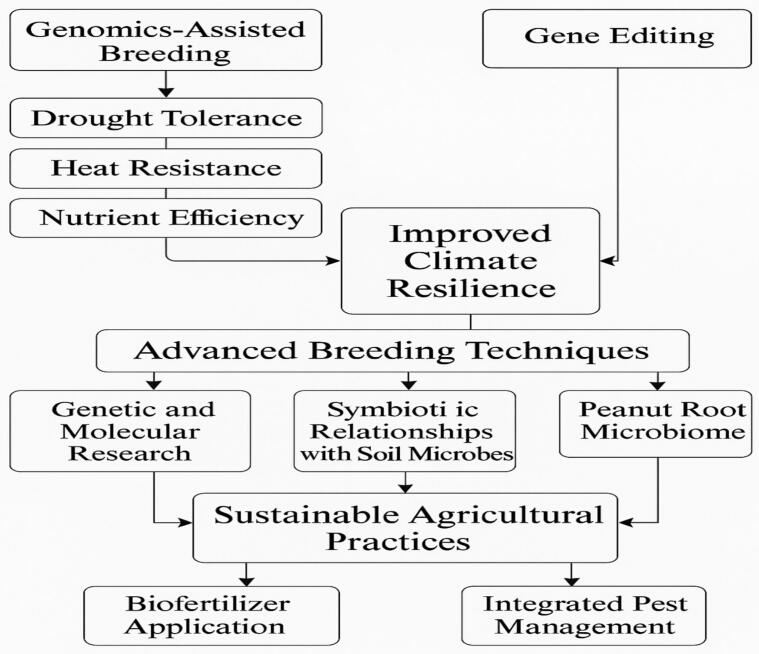
Future directions in peanut research and climate adaptation.

Future peanut research for climate adaptation is focused on developing more resilient varieties using advanced breeding techniques, including genomics-assisted breeding and gene editing technologies like CRISPR. Researchers are delving deeper into the genetic foundation of traits such as drought tolerance, heat resistance, and nutrient efficiency, with the goal of incorporating these traits into high-yielding peanut cultivars (Fig. 5). In addition to genetic improvements, there is a growing emphasis on sustainable agricultural practices that enhance soil health, such as biofertilizer application and integrated pest management, which collectively contribute to improved climate resilience. Research is also expanding into the peanut root microbiome, exploring how symbiotic relationships with soil microbes can improve nutrient uptake and stress tolerance. By combining genetic, molecular, and agronomic strategies, these efforts hold great promise for maintaining sustainable and productive peanut production despite the challenges posed by climate change.

## 9 Conclusion and recommendation

In conclusion, root system architecture is a foundational determinant of peanut resilience under climate-induced stress, particularly drought and nutrient-deficient soils. Deep, efficient, and highly branched root systems enhance the plant’s capacity to access subsoil resources, directly contributing to improved physiological performance under environmental constraints. Advances in root phenotyping, combined with molecular breeding strategies targeting traits such as root depth, lateral root density, and xylem efficiency, offer tangible pathways for varietal improvement. Complementary agronomic practices including conservation tillage, mulching, and diversified crop rotations can synergistically enhance root development and function by improving soil structure and moisture retention. Looking ahead, a systems-level approach integrating genomics, transcriptomics, and microbiome profiling with ecologically sound field practices will be essential for developing climate-resilient peanut cultivars. Harnessing root exudates to foster beneficial microbial communities complements traditional breeding for root architecture, enhancing peanut resilience under diverse climate stresses. Integrating microbiome-focused traits with molecular breeding expands the potential for improved nutrient acquisition and stress tolerance. Broadening focus beyond drought to include heat and salinity will ensure more comprehensive climate adaptability. Equally important is supporting farmer adoption through locally tailored agronomic practices to realize sustainable impact. Such multidimensional strategies will not only strengthen stress adaptation but also support long-term agricultural sustainability and food security in a rapidly changing climate.

**Data availability**

Data sharing is not applicable to this article as no new data were generated or analyzed.

## Funding statement

This work was supported by the National Natural Science Foundation of China (Grant No. 32171997), the Earmarked Fund for China Agriculture Research System (CARS-13), the Nanfan special project of CAAS (Grant No. YBXM2552), the Central Public-interest Scientific Institution Basal Research Fund (Grant No. Y2025YC112), and the Agricultural Science and Technology Innovation Program of the Chinese Academy of Agricultural Sciences (Grant No. CAAS-ASTIP-2021-OCRI).

## CRediT authorship contribution statement

**Yohannes Gelaye:** Writing – review & editing, Writing – original draft, Visualization, Investigation, Formal analysis, Data curation, Conceptualization. **Jihua Li:** Writing – review & editing. **Huaiyong Luo:** Writing – review & editing, Validation, Supervision, Project administration.

## Declaration of competing interest

The authors declare the following financial interests/personal relationships which may be considered as potential competing interests: Yohannes Gelaye reports administrative support was provided by Debre Markos University College of Agriculture and Natural Resource. Yohannes Gelaye reports a relationship with Debre Markos University College of Agriculture and Natural Resource that includes: employment. Yohannes Gelaye has patent licensed to Licensed. There is no any relationship to be disclose. If there are other authors, they declare that they have no known competing financial interests or personal relationships that could have appeared to influence the work reported in this paper.

## References

[1] Sharma K.K., Bhatnagar-Mathur P. (2006). Peanut (*Arachis hypogaea* L.). Agrobacterium Protocols.

[2] Dwivedi S., Crouch J., Nigam S., Ferguson M., Paterson A. (2003). Molecular breeding of groundnut for enhanced productivity and food security in the semi-arid tropics: opportunities and challenges. Adv Agron.

[3] Puppala N., Nayak S.N., Sanz-Saez A. (2023). Sustaining yield and nutritional quality of peanuts in harsh environments: Physiological and molecular basis of drought and heat stress tolerance. Front Genet.

[4] Meisner C., Karnok K. (1992). Peanut root response to drought stress. Agron J.

[5] Lynch J. (1995). Root architecture and plant productivity. Plant Physiol.

[6] Seitz V.A., McGivern B.B., Borton M.A. (2024). Cover crop root exudates impact soil microbiome functional trajectories in agricultural soils. Microbiome.

[7] Lyu D., Smith D.L. (2022). The root signals in rhizospheric inter-organismal communications. Front Plant Sci.

[8] Singh S.S., Singh N.S., Lamare E. (2025). Drought mitigation in plants through root exudate-mediated rhizosphere interactions: Opportunities for future research. Curr Plant Biol.

[9] Li X., Jousset A., de Boer W. (2019). Legacy of land use history determines reprogramming of plant physiology by soil microbiome. ISME J.

[10] Chen W, Modi D, Picot A. Soil and phytomicrobiome for plant disease suppression and management under climate change: a review. Plants (Basel) 2023, 12. 10.3390/plants12142736 PMC10384710 37514350

[11] George T.S., Bulgarelli D., Carminati A. (2024). Bottom-up perspective – the role of roots and rhizosphere in climate change adaptation and mitigation in agroecosystems. Plant and Soil.

[12] Park J.-W., Braswell W.E., Kunta M. (2024). Co-occurrence analysis of citrus root bacterial microbiota under citrus greening disease. Plants.

[13] Li L., Li Q., Davis K.E. (2021). Response of root growth and development to nitrogen and potassium deficiency as well as microRNA-mediated mechanism in peanut (*Arachis hypogaea* L.).. Front Plant Sci.

[14] Janila P., Variath M.T., Pandey M.K. (2016). Genomic tools in groundnut breeding program: status and perspectives. Front Plant Sci.

[15] Pandey M.K., Monyo E., Ozias-Akins P. (2012). Advances in Arachis genomics for peanut improvement. Biotechnol Adv.

[16] Thangthong N., Jogloy S., Jongrungklang N. (2018). Root distribution patterns of peanut genotypes with different drought resistance levels under early‐season drought stress. J Agron Crop Sci.

[17] Patel J., Khandwal D., Choudhary B. (2022). Differential physio-biochemical and metabolic responses of peanut (*Arachis hypogaea* L.) under multiple abiotic stress conditions. Int J Mol Sci.

[18] Reddy T., Reddy V., Anbumozhi V. (2003). Physiological responses of groundnut (*Arachis hypogea* L.) to drought stress and its amelioration: a critical review. Plant Growth Regul.

[19] Singh A. (2004). Growth and physiology of groundnut. Groundnut Research in India.

[20] Rachaputi R, Chauhan YS, Wright GC: Peanut. Crop physiology case histories for major crops: Elsevier, 2021. pp. 360-82.

[21] Thangthong N., Jogloy S., Pensuk V., Kesmala T., Vorasoot N. (2016). Distribution patterns of peanut roots under different durations of early season drought stress. Field Crop Res.

[22] Kalariya K., Singh A., Nakar R., Zala P., Chakraborty K., Patel C. (2018). Physiological plasticity of Arachis hypogaea cultivars under drought conditions of semiarid region of India. J Food Legumes.

[23] Zhang W., Wang X.-X., Yang Z. (2017). Physiological mechanisms behind endophytic fungus Phomopsis liquidambari-mediated symbiosis enhancement of peanut in a monocropping system. Plant and Soil.

[24] Geiss G, Gutierrez L, Bellini C: Adventitious root formation: new insights and perspectives. Annual plant reviews volume 37: root development 2009, 37:127-56.

[25] Zuo Y., Li X., Cao Y., Zhang F., Christie P. (2003). Iron nutrition of peanut enhanced by mixed cropping with maize: possible role of root morphology and rhizosphere microflora. J Plant Nutr.

[26] Zhang J., Elliott R., Ketring D. (1993). Root distribution models applied to peanuts. Trans ASAE.

[27] Micucci C. (2000).

[28] Ketring D., Jordan W., Smith O., Simpson C. (1982). Genetic variability in root and shoot growth characteristics of peanut. Peanut Science.

[29] Jackson R.B., Pockman W.T., Hoffmann W.A., Bleby T.M., Armas C. (2007).

[30] Morgante C., Angelini J., Castro S., Fabra A. (2005). Role of rhizobial exopolysaccharides in crack entry/intercellular infection of peanut. Soil Biol Biochem.

[31] Grönemeyer J.L., Chimwamurombe P., Reinhold-Hurek B. (2015). Bradyrhizobium subterraneum sp. nov., a symbiotic nitrogen-fixing bacterium from root nodules of groundnuts. Int J Syst Evol Microbiol.

[32] Noorhosseini S.A., Soltani A., Ajamnoroozi H. (2017). Modeling the impact of climate change on peanut production on the basis of increasing 2oc temperature in future environmental conditions of Guilan Province, Iran. Int J Agric Manage Dev (IJAMAD).

[33] Vara Prasad P., Boote K.J., Hartwell Allen Jr L., Thomas J.M. (2003). Super‐optimal temperatures are detrimental to peanut (*Arachis hypogaea* L.) reproductive processes and yield at both ambient and elevated carbon dioxide. Glob Chang Biol.

[34] Quilambo O.A. (2000). Under Nutrient Deficiency and Drought Stress in Relation to Symbiotic Associations.

[35] Ruane A.C., McDermid S., Rosenzweig C. (2014). Carbon–temperature–water change analysis for peanut production under climate change: a prototype for the AgMIP Coordinated Climate‐Crop Modeling Project (C3 MP). Glob Chang Biol.

[36] Thoppurathu F.J., Ghorbanzadeh Z., Vala A.K., Hamid R., Joshi M. (2022). Unravelling the treasure trove of drought-responsive genes in wild-type peanut through transcriptomics and physiological analyses of root. Funct Integr Genomics.

[37] Rao R.N., Sheshshayee M., Karaba N.N. (2012). Genetic approaches to enhance adaptation of groundnut (*Arachis Hypogaea* L.) to drought. Improving Crop Productivity in Sustainable Agriculture.

[38] Wang J., Yu Y., Jiang C. (2023). Comparative analysis of physiology-anatomy and transcriptome-metabolome involving acute drought stress response of root between two distinct peanut cultivars at seedling stage. Environ Exp Bot.

[39] Banavath J.N., Chakradhar T., Pandit V. (2018). Stress inducible overexpression of AtHDG11 leads to improved drought and salt stress tolerance in peanut (*Arachis hypogaea* L.). Front Chem.

[40] Dai L., Zhang G., Yu Z., Ding H., Xu Y., Zhang Z. (2019). Effect of drought stress and developmental stages on microbial community structure and diversity in peanut rhizosphere soil. Int J Mol Sci.

[41] Fabra A., Castro S., Taurian T. (2010). Interaction among Arachis hypogaea L.(peanut) and beneficial soil microorganisms: how much is it known?. Crit Rev Microbiol.

[42] Ankati S., Rani T.S., Podile A.R. (2019). Changes in root exudates and root proteins in groundnut–Pseudomonas sp. interaction contribute to root colonization by bacteria and defense response of the host. J Plant Growth Regul.

[43] Sarraf M., Janeeshma E., Arif N. (2023). Understanding the role of beneficial elements in developing plant stress resilience: Signalling and crosstalk with phytohormones and microbes. Plant Stress.

[44] Iqbal S., Wang X., Mubeen I. (2022). Phytohormones trigger drought tolerance in crop plants: outlook and future perspectives. Front Plant Sci.

[45] Calleja-Cabrera J., Boter M., Oñate-Sánchez L., Pernas M. (2020). Root growth adaptation to climate change in crops. Front Plant Sci.

[46] Gelaye Y., Luo H. (2024). Optimizing peanut (*Arachis hypogaea* L.) production: genetic insights, climate adaptation, and efficient management practices: systematic review. Plants.

[47] Janiak A., Kwaśniewski M., Szarejko I. (2016). Gene expression regulation in roots under drought. J Exp Bot.

[48] Ganie S.A., Ahammed G.J. (2021). Dynamics of cell wall structure and related genomic resources for drought tolerance in rice. Plant Cell Rep.

[49] Joshi S., Nath J., Singh A.K., Pareek A., Joshi R. (2022). Ion transporters and their regulatory signal transduction mechanisms for salinity tolerance in plants. Physiol Plant.

[50] López-Maury L., Marguerat S., Bähler J. (2008). Tuning gene expression to changing environments: from rapid responses to evolutionary adaptation. Nat Rev Genet.

[51] Hu B: Ling li (2019). Arachis hypogaea histone deacetylase 1 (AhHDA1) is involved in the epigenetic regulation of the drought stress response. of, 4:2.

[52] Li P., Peng Z., Xu P. (2021). Genome-wide identification of NAC transcription factors and their functional prediction of abiotic stress response in peanut. Front Genet.

[53] Ping X., Ye Q., Yan M. (2024). Overexpression of BnaA10. WRKY75 decreases cadmium and salt tolerance via increasing ROS accumulation in Arabidopsis and Brassica napus L. Int J Mol Sci.

[54] Li C., Yan C., Sun Q. (2022). Proteomic profiling of Arachis hypogaea in response to drought stress and overexpression of AhLEA2 improves drought tolerance. Plant Biol.

[55] Aravind B., Shreeraksha R., Poornima R. (2024). Impact of heat stress on physiological characteristics and expression of heat shock proteins (HSPs) in groundnut (Arachis hypogaea L.). Physiol Mol Biol Plants.

[56] Hwarari D., Guan Y., Ahmad B. (2022). ICE-CBF-COR signaling cascade and its regulation in plants responding to cold stress. Int J Mol Sci.

[57] Long HaiTao LH, Zheng Zhao ZZ, Zhang YaJun ZY, Xing PengZhan XP, Wan XiaoRong WX, Zheng YiXiong ZY, Li Ling LL: An abscisic acid (ABA) homeostasis regulated by its production, catabolism and transport in peanut leaves in response to drought stress; 2019. 10.1371/journal.pone.0213963 PMC6594590 31242187

[58] Long H., Zheng Z., Zhang Y. (2019). An abscisic acid (ABA) homeostasis regulated by its production, catabolism and transport in peanut leaves in response to drought stress. PLoS One.

[59] Garg G., Neha P. (2019). Plant transcription factors networking of pyrroline-5-carboxylate (P5C) enzyme under stress condition: a review. Plant Arch.

[60] Liu Y., Shen Y., Liang M. (2022). Identification of peanut AhMYB44 transcription factors and their multiple roles in drought stress responses. Plants.

[61] Wang F., Yang F., Zhu D., Saniboere B., Zhou B., Peng D. (2024). MYB44 plays key roles in regulating plant responses to abiotic and biotic stress, metabolism, and development. J Plant Biochem Biotechnol.

[62] Wu J., Zhang N., Liu Z. (2020). The AtGSTU7 gene influences glutathione-dependent seed germination under ABA and osmotic stress in Arabidopsis. Biochem Biophys Res Commun.

[63] Yang X., Lu M., Wang Y., Wang Y., Liu Z., Chen S. (2021). Response mechanism of plants to drought stress. Horticulturae.

[64] Atta K., Mondal S., Gorai S. (2023). Impacts of salinity stress on crop plants: improving salt tolerance through genetic and molecular dissection. Front Plant Sci.

[65] Liu Y., Shen Y., Liang M. (2022). Identification of peanut AhMYB44 transcription factors and their multiple roles in drought stress responses. Plants (Basel).

[66] Satyakam Z.G., Singh R.K., Kumar R. (2022). Cold adaptation strategies in plants—An emerging role of epigenetics and antifreeze proteins to engineer cold resilient plants. Front Genet.

[67] Zhang B., Su L., Hu B., Li L. (2018). Expression of AhDREB1, an AP2/ERF transcription factor gene from peanut, is affected by histone acetylation and increases abscisic acid sensitivity and tolerance to osmotic stress in arabidopsis. Int J Mol Sci.

[68] Banerjee Mustafi S., Chakraborty P.K., Dey R.S., Raha S. (2009). Heat stress upregulates chaperone heat shock protein 70 and antioxidant manganese superoxide dismutase through reactive oxygen species (ROS), p38MAPK, and Akt. Cell Stress Chaperones.

[69] Gangurde S.S., Kumar R., Pandey A.K. (2019). Climate-smart groundnuts for achieving high productivity and improved quality: current status, challenges, and opportunities. Genomic Designing of Climate-Smart Oilseed Crops.

[70] Hamidou F., Halilou O., Vadez V. (2013). Assessment of groundnut under combined heat and drought stress. J Agron Crop Sci.

[71] Kar G., Kumar A. (2007). Surface energy fluxes and crop water stress index in groundnut under irrigated ecosystem. Agric For Meteorol.

[72] Qin F, Xin Z, Wang J, Zhang J, Yang J, Guo F, Ci D. Peanut production in saline-alkali land of Yellow River Delta: influence of spatiotemporal changes of meteorological conditions and soil properties; 2024. 10.1186/s12870-024-05745-7 PMC11524010 39472776

[73] Aydinşakir K., Büyüktaş D., Dinç N., Karaca C. (2015). Impact of salinity stress on growing, seedling development and water consumption of peanut (*Arachis hypogaea* cv. NC-7). Akdeniz Univ J Faculty Agric.

[74] Barzana G., Rios J.J., Lopez-Zaplana A. (2021). Interrelations of nutrient and water transporters in plants under abiotic stress. Physiol Plant.

[75] Hussain Q., Asim M., Zhang R., Khan R., Farooq S., Wu J. (2021). Transcription factors interact with ABA through gene expression and signaling pathways to mitigate drought and salinity stress. Biomolecules.

[76] Huang G., Zhang D. (2020). The plasticity of root systems in response to external phosphate. Int J Mol Sci.

[77] Yu P., He X., Baer M. (2021). Plant flavones enrich rhizosphere Oxalobacteraceae to improve maize performance under nitrogen deprivation. Nat Plants.

[78] Liu Y., Shao L., Zhou J. (2022). Genomic insights into the genetic signatures of selection and seed trait loci in cultivated peanut. J Adv Res.

[79] Jiang H.-J., Zhao Y.-Y., Pan Y.-T., Sun K., Xie X.-G., Dai C.-C. (2022). The endophytic fungus Phomopsis liquidambaris promotes phosphorus uptake by *Arachis hypogaea* L. by regulating host auxin, gibberellins, and cytokinins signaling pathways. J Soil Sci Plant Nutr.

[80] Zhang Z., Chen N., Zhang Z., Shi G. (2022). Genome-wide identification and expression profile reveal potential roles of peanut ZIP family genes in zinc/iron-deficiency tolerance. Plants.

[81] Filiz E., Akbudak M.A. (2020). Ammonium transporter 1 (AMT1) gene family in tomato (*Solanum lycopersicum* L.): Bioinformatics, physiological and expression analyses under drought and salt stresses. Genomics.

[82] Omari Alzahrani F. (2025). Ammonium transporter 1 (AMT1) gene family in pomegranate: genome-wide analysis and expression profiles in response to salt stress. Curr Issues Mol Biol.

[83] Estrada Y., Fernández-Ojeda A., Morales B. (2021). Unraveling the strategies used by the underexploited amaranth species to confront salt stress: Similarities and differences with quinoa species. Front Plant Sci.

[84] Li Z., Cao Z., Ma X. (2024). Natural resistance-associated macrophage proteins are involved in tolerance to heavy metal Cd2+ toxicity and resistance to bacterial wilt of peanut (*Arachis hypogaea* L.).. Plant Physiol Biochem.

[85] Yan L., Jin H., Raza A., Huang Y., Dp Gu, Zou X. (2024). Natural resistance-associated macrophage proteins (NRAMPs) are involved in cadmium enrichment in peanut (*Arachis hypogaea* L.) under cadmium stress. Plant Growth Regul.

[86] Zia R., Nawaz M.S., Siddique M.J., Hakim S., Imran A. (2021). Plant survival under drought stress: Implications, adaptive responses, and integrated rhizosphere management strategy for stress mitigation. Microbiol Res.

[87] Hao S., Wang Y., Yan Y., Liu Y., Wang J., Chen S. (2021). A review on plant responses to salt stress and their mechanisms of salt resistance. Horticulturae.

[88] Zhang W.D., Wang P., Bao Z. (2017). SOS1, HKT1;5, and NHX1 synergistically modulate Na(+) homeostasis in the halophytic grass Puccinellia tenuiflora. Front Plant Sci.

[89] Rao Y.R., Ansari M.W., Sahoo R.K., Wattal R.K., Tuteja N., Kumar V.R. (2021). Salicylic acid modulates ACS, NHX1, sos1 and HKT1; 2 expression to regulate ethylene overproduction and Na+ ions toxicity that leads to improved physiological status and enhanced salinity stress tolerance in tomato plants cv. Pusa Ruby. Plant Signaling & Behavior.

[90] Luo M., Chu J., Wang Y., Chang J., Zhou Y., Jiang X. (2024). Positive regulatory roles of Manihot esculenta HAK5 under K+ deficiency or high salt stress. Plants.

[91] Zhang J., Wei J., Li D. (2017). The role of the plasma membrane H+-ATPase in plant responses to aluminum toxicity. Front Plant Sci.

[92] Qari S.H., Hassan M.U., Chattha M.U. (2022). Melatonin induced cold tolerance in plants: physiological and molecular responses. Front Plant Sci.

[93] Zheng D., Han X., An Y.I., Guo H., Xia X., Yin W. (2013). The nitrate transporter NRT2.1 functions in the ethylene response to nitrate deficiency in Arabidopsis. Plant Cell Environ.

[94] Çetin D., Akbudak M.A. (2024). Cold stress impairs nitrogen uptake and enhances translocation through AMT1 and NRT2 gene regulation in tomato. Mediterranean Agricultural Sciences.

[95] Patra A.K., Kar I. (2021). Heat stress on microbiota composition, barrier integrity, and nutrient transport in gut, production performance, and its amelioration in farm animals. J Anim Sci Technol.

[96] Gomez-Pastor R., Burchfiel E.T., Thiele D.J. (2018). Regulation of heat shock transcription factors and their roles in physiology and disease. Nat Rev Mol Cell Biol.

[97] Mishra S.K., Tripp J., Winkelhaus S. (2002). In the complex family of heat stress transcription factors, HsfA1 has a unique role as master regulator of thermotolerance in tomato. Genes Dev.

[98] Luo L., Wan Q., Yu Z. (2022). Genome-wide identification of auxin response factors in peanut (*Arachis hypogaea* L.) and functional analysis in root morphology. Int J Mol Sci.

[99] de Dorlodot S., Forster B., Pagès L., Price A., Tuberosa R., Draye X. (2007). Root system architecture: opportunities and constraints for genetic improvement of crops. Trends Plant Sci.

[100] Kumar N. Leveraging genomic mapping and QTL analysis to enhance drought tolerance of cultivated peanut (*Arachis hypogaea* L.); 2022.

[101] Zhao N., Cui S., Li X. (2021). Transcriptome and co-expression network analyses reveal differential gene expression and pathways in response to severe drought stress in peanut (*Arachis hypogaea* L.). Front Genet.

[102] Kalarani M., Senthil A., Punitha S., Sowmyapriya S., Umapathi M., Geethalakshmi V. (2023). Abiotic stress responses in groundnut (*Arachis hypogaea* L.): mechanisms and adaptations. Legumes: Physiol. Mol. Biol. Abiotic Stress Tolerance: Springer.

[103] Novaković L., Guo T., Bacic A., Sampathkumar A., Johnson K.L. (2018). Hitting the wall—Sensing and signaling pathways involved in plant cell wall remodeling in response to abiotic stress. Plants.

[104] Ranjan A., Sinha R., Singla-Pareek S.L., Pareek A., Singh A.K. (2022). Shaping the root system architecture in plants for adaptation to drought stress. Physiol Plant.

[105] Koevoets I.T., Venema J.H., Elzenga J.T.M., Testerink C. (2016). Roots withstanding their environment: exploiting root system architecture responses to abiotic stress to improve crop tolerance. Front Plant Sci.

[106] Li L., Li Q., Liu Y. (2024). Diversity, variance, and stability of root phenes of peanut (*Arachis hypogaea* L.).. Physiol Plant.

[107] Xiao D., Li X., Zhou Y.-Y. (2021). Transcriptome analysis reveals significant difference in gene expression and pathways between two peanut cultivars under Al stress. Gene.

[108] Tarkowski Ł.P., Signorelli S., Considine M.J., Montrichard F. (2023). Integration of reactive oxygen species and nutrient signalling to shape root system architecture. Plant Cell Environ.

[109] Kumar M., Rani K. (2023). Epigenomics in stress tolerance of plants under the climate change. Mol Biol Rep.

[110] McGaughey S.A., Qiu J., Tyerman S.D., Byrt C.S. (2018). Regulating root aquaporin function in response to changes in salinity. Annual Plant Reviews Online.

[111] Zanetti M.E., Rípodas C., Niebel A. (2017). Plant NF-Y transcription factors: Key players in plant-microbe interactions, root development and adaptation to stress. Biochim Biophys Acta (BBA)-Gene Regul Mech.

[112] Ezquer I., Salameh I., Colombo L., Kalaitzis P. (2020). Plant cell walls tackling climate change: Insights into plant cell wall remodeling, its regulation, and biotechnological strategies to improve crop adaptations and photosynthesis in response to global warming. Plants.

[113] Joshi S., Chinnusamy V., Joshi R. (2022). Root system architecture and omics approaches for belowground abiotic stress tolerance in plants. Agriculture.

[114] Tiwari P., Srivastava D., Chauhan A.S. (2021). Root system architecture, physiological analysis and dynamic transcriptomics unravel the drought-responsive traits in rice genotypes. Ecotoxicol Environ Saf.

[115] Gundaraniya S.A., Ambalam P.S., Tomar R.S. (2020). Metabolomic profiling of drought-tolerant and susceptible peanut (*Arachis hypogaea* L.) genotypes in response to drought stress. ACS Omega.

[116] Carrera F.P., Noceda C., Maridueña-Zavala M.G., Cevallos-Cevallos J.M. (2021). Metabolomics, a powerful tool for understanding plant abiotic stress. Agronomy.

[117] Xue A., Cui Y. (2025). The research progress on crop genomics and genome-wide association studies: a review. Adv Resour Res.

[118] Jiang C., Li X., Zou J. (2021). Comparative transcriptome analysis of genes involved in the drought stress response of two peanut (*Arachis hypogaea* L.) varieties. BMC Plant Biol.

[119] Dong X., Gao Y., Bao X. (2023). Multi-omics revealed Peanut root metabolism regulated by exogenous calcium under salt stress. Plants.

[120] Guo X., Lv L., Zhao A. (2025). Integrated transcriptome and metabolome analysis revealed differential drought stress response mechanisms of wheat seedlings with varying drought tolerance. BMC Plant Biol..

[121] Zhang K., Qin Y., Sun W. (1944). Phylogenomic analysis of cytochrome P450 gene superfamily and their association with flavonoids biosynthesis in peanut (*Arachis hypogaea* L.). Genes.

[122] Wan L., Lei Y., Yan L. (2020). Transcriptome and metabolome reveal redirection of flavonoids in a white testa peanut mutant. BMC Plant Biol.

[123] Wang W., Liu C., Du S., Zang C.-Q., Huang Y.-Q. (2024). Metabolic response of peanut (*Arachis hypogaea* L.) to Sclerotium rolfsii Sacc. in root exudates system. J Plant Interact.

[124] Yang D., Liu Y., Wang Y. (2020). Effects of soil tillage, management practices, and mulching film application on soil health and peanut yield in a continuous cropping system. Front Microbiol.

[125] Cao T., Zang X., Ren J., Liu J., Yang D. (2024). Cover crop alters rhizosphere sediments to recruit plant growth-promoting microorganisms, enhancing peanut production. Appl Soil Ecol.

[126] Wann D.Q. (2011).

[127] El-Beltagi H.S., Basit A., Mohamed H.I. (1881). Mulching as a sustainable water and soil saving practice in agriculture: a review. Agronomy.

[128] Wright D., Marois J., Rich J., Sprenkel R., Whitty E. (2002). University of Florida Institute of Food and Agricultural Sciences SS-AGR-185.

[129] Zhen X., Zhang Q., Sanz-Saez A., Chen C.Y., Dang P.M., Batchelor W.D. (2022). Simulating drought tolerance of peanut varieties by maintaining photosynthesis under water deficit. Field Crop Res.

[130] Zou X., Liu Y., Huang M. (2023). Rotational strip intercropping of maize and peanut enhances productivity by improving crop photosynthetic production and optimizing soil nutrients and bacterial communities. Field Crop Res.

[131] Songsri P., Jogloy S., Vorasoot N., Akkasaeng C., Patanothai A., Holbrook C. (2008). Root distribution of drought‐resistant peanut genotypes in response to drought. J Agron Crop Sci.

[132] Halilou O., Hamidou F., Taya B.K., Mahamane S., Vadez V. (2015). Water use, transpiration efficiency and yield in cowpea (*Vigna unguiculata*) and peanut (*Arachis hypogaea* L.) across water regimes. Crop Pasture Sci.

[133] Lai H., Li X., Chen Y., Liu Z. (2024). Mitigating heat-induced yield loss in peanut: Insights into 24-epibrassinolide-mediated improvement in antioxidant capacity, photosynthesis, and kernel weight. Field Crop Res.

[134] Van Chuong N. The impact of bacillus sp. NTLG2-20 and reduced nitrogen fertilization on soil properties and peanut yield. Commun Sci Technol 2024, 9:112-20.

[135] Kambiranda D.M., Vasanthaiah H.K., Katam R., Ananga A., Basha S.M., Naik K. (2011). Impact of drought stress on peanut (*Arachis hypogaea* L.) productivity and food safety. Plants and Environment.

[136] Anyia A., Herzog H. (2004). Water-use efficiency, leaf area and leaf gas exchange of cowpeas under mid-season drought. Eur J Agron.

[137] Singh A. (2011). Physiological basis for realizing yield potentials in groundnut. Adv Plant Physiol.

[138] Akimoto M., Sato S., Tanaka I. (2024). The influence of planting density on the flowering pattern and seed yield in peanut (*Arachis hypogea* L.) grown in the northern region of Japan. Agriculture.

[139] Ndoye O., Smith O. (1992). Flowering pattern and fruiting characteristics of five short growth duration peanut lines. Oleagineux.

